# Interacting Effects Induced by Two Neighboring Pits Considering Relative Position Parameters and Pit Depth

**DOI:** 10.3390/ma10040398

**Published:** 2017-04-09

**Authors:** Yongfang Huang, Tieqiang Gang, Lijie Chen

**Affiliations:** School of Aerospace Engineering, Xiamen University, Xiamen 361005, Fujian, China; chenlijie@xmu.edu.cn

**Keywords:** pitting corrosion, stress concentration factor, finite element analysis, relative position parameters, pit depth

## Abstract

For pre-corroded aluminum alloy 7075-T6, the interacting effects of two neighboring pits on the stress concentration are comprehensively analyzed by considering various relative position parameters (inclination angle *θ* and dimensionless spacing parameter *λ*) and pit depth (*d*) with the finite element method. According to the severity of the stress concentration, the critical corrosion regions, bearing high susceptibility to fatigue damage, are determined for intersecting and adjacent pits, respectively. A straightforward approach is accordingly proposed to conservatively estimate the combined stress concentration factor induced by two neighboring pits, and a concrete application example is presented. It is found that for intersecting pits, the normalized stress concentration factor *K*_tnor_ increases with the increase of *θ* and *λ* and always reaches its maximum at *θ* = 90°, yet for adjacent pits, *K*_tnor_ decreases with the increase of *λ* and the maximum value appears at a slight asymmetric location. The simulations reveal that *K*_tnor_ follows a linear and an exponential relationship with the dimensionless depth parameter *R*_d_ for intersecting and adjacent cases, respectively.

## 1. Introduction

Pitting corrosion is considered to be one of the most significant degradation mechanisms as it causes stress concentration and facilitates crack initiation, thus accelerating structural failure under fatigue loading conditions [1,2,3]. Therefore, understanding the stress environment around corrosion pits can assist the early prediction of critical regions bearing a relatively high probability of crack initiation, which is of particular significance to maintain structural integrity, especially for aircrafts, offshore structures, and other applications with high criticality or low tolerance of unwanted degradation.

It is believed that pit size, location, geometry, and the interaction with other pits are dominant factors which affect the stress concentration. A number of studies have been conducted to discuss the influence of the first three factors [4,5,6,7,8,9,10], yet limited research has focused on the interacting effects induced by neighboring pits. Domínguez et al. [11] examined the stress concentration for two close pitting holes located on the hourglass-shape specimen of aluminum alloy 6061-T6, and found that the stress concentration factor (SCF) increased exponentially with the proximity of pitting holes under the rotating-bending loading condition. Han et al. [12] studied the mechanical property of transmission pipelines with inner corrosion defects via the finite element method. Results showed that for the pipeline with two or three longitudinally-aligned corrosion defects, high stress areas and the plastic strain increased rapidly along the axial direction with the increasing of the inner pressure. By conducting 2D simulations, Kolios et al. [13] revealed that a lower spacing between pits resulted in a higher SCF, and colluded pits had a smaller stress-concentrating effect than pits spaced at a larger distance from each other. Hou et al. [14] discussed the stress concentration induced by two adjacent pits under a uniaxial tension loading condition. They pointed out that the SCF decreased nonlinearly with the increasing of the spacing between two transverse pits, and the interaction between two longitudinal pits should be ignored in the SCF calculation. Pidaparti et al. [15] predicted the stress distribution on a corroded random surface and found that the single pit induced a stress about 70% higher than the two-pit case for a 74-day corroded specimen. Xu [16] investigated the tensile behavior of eight different-depth spherical pits equally distributed at four lines along the axial direction on the reinforcement surface, and emphasized that the most disadvantageous location coincided with the deepest pit. The above-mentioned studies, however, mainly focus on neighboring pits aligned along transverse and/or longitudinal directions, which is obviously insufficient when comparing to the random spatial distribution of pitting corrosion in the actual environment. Furthermore, the influence of the pit depth on interacting effects has scarcely been taken into account in the available publications.

In our previous works [17,18], high-cycle fatigue tests were conducted for the pre-corroded aluminum alloy (AA) 7075-T6 specimens. Fracture analysis showed that under 120 h and 240 h pre-corrosion conditions, 64.9% (24 out of 37 pieces) and 44.1% (15 out of 34 pieces) of the specimens respectively failed due to the propagation of fatigue cracks originating at two neighboring pits. This is considered to be an interesting phenomenon and deserved further investigation.

In this work, for pre-corroded AA7075-T6 specimens, numerical simulations are carried out to investigate how a neighboring pit will exert influence on the stress field caused by a single pit, and how the critical corrosion region is identified for two intersecting and adjacent pits, respectively. By synthetically considering the pit depth and the relative position parameters, a straightforward approach is proposed to conservatively estimate the combined stress concentration factor (C-SCF) induced by two neighboring pits.

## 2. Finite Element Model

**Figure 1:**
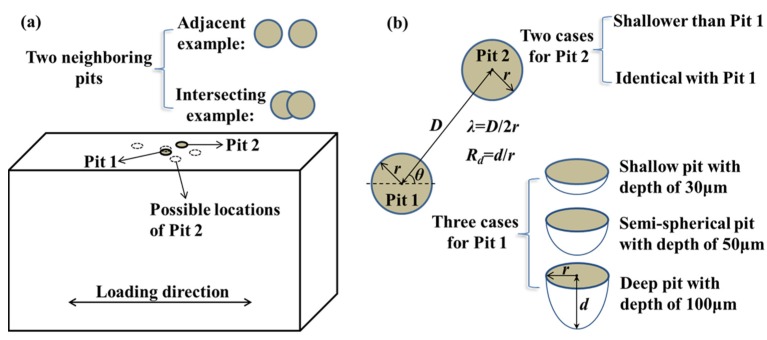
Details of the model containing two neighboring pits. (**a**) Sketch outline of the adjacent and intersecting cases; and (**b**) diagram of the configurations of Pit 1 and Pit 2.

Details of the model for two neighboring pits are illustrated in Figure 1. The geometry involves a cuboid (10 mm × 6 mm × 2 mm) containing Pit 1 and Pit 2 on its top surface. The actual pit is idealized with a hemi-elliptical shape and is assumed to be capable of representing the stress field around the actual pit [19]. In order to examine the effect of the spatial distribution on the C-SCF, Pit 1 is placed at the center of the gage area as a reference for comparison, while Pit 2 is located at a random location on the top surface. Thus, the arrangements of Pit 1 and Pit 2, according to their relative positions, can be classified into two cases: adjacent pits and intersecting pits, as exemplified in Figure 1a.

Two relative position parameters, *θ* and *λ*, are introduced to quantitatively describe the relative locations of Pit 1 and Pit 2, which is illustrated in Figure 1b by taking two adjacent pits as an example. Here, *θ* is defined as the inclination angle between the loading direction and the line connecting centers of the two pits, and *λ* as a dimensionless parameter is used to measure the proximity of the two pits and is defined below,

*λ* = *D*/2*r* (1)

where *D* is the spacing between the centers of Pit 1 and Pit 2, and *r* the radius for both pits, which is assigned to be 50 μm in our study. To clarify the influence of the pit depth (*d*) on interacting effects, we configure the depth of Pit 1 (*d*_1_) with three settings: 30 μm, 50 μm, and 100 μm, respectively, which correspondingly represent shallow, hemi-spherical, and deep pits. For Pit 2, we consider two cases of the pit depth (*d*_2_), which is either shallower than (*d*_2_ < *d*_1_) or identical with (*d*_2_ = *d*_1_ = *d*) that of Pit 1. The specific definitions and settings for all the configurations of Pit 1 and Pit 2 are listed in Table 1. In our simulations, the geometrical dimensions are referenced to our previous testing results [17,18,20], i.e., the pit width and the aspect ratio (depth to half width) are in the range of 2–130 μm and 0.4–4.8, respectively. Additionally, in consideration of other pit shapes reported in the literature, such as dish-shaped [21] and needle-shaped pits [22], our FEA (finite element analysis) models are run within a relatively wide range of parameters to improve the validity and applicability.

**Figure 2:**
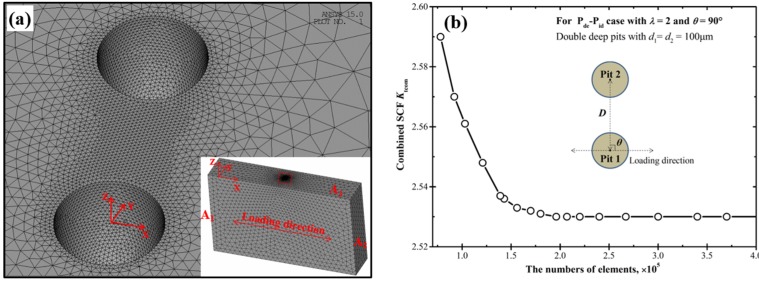
The results of finite element meshing and the convergence study. (**a**) The mesh refinement around the pits with the inset showing the whole model meshes; and (**b**) the convergence study with different numbers of elements.

Simulations here are performed within the linear elastic framework since all our fatigue tests in previous works [17,18] were conducted within the range of linear elasticity. The stress concentration factor, defined as the ratio of the local maximum stress to the far field stress (here it is a uniformly distributed area load of 1 MPa), is analyzed by using ANSYS software in the range of 0 < *λ* ≤ 2.5 and 0 ≤ *θ* ≤ 90° for all the cases of Pit 1 and Pit 2. Owing to the symmetry of the spatial distribution, the results for 0 ≤ *θ* ≤ 90° can fully represent those for 0 ≤ *θ* ≤ 360°. So our discussions only focus on the range of 0 ≤ *θ* ≤ 90°. Due to the inner curved-surface of the pits, and especially for the irregular shapes caused by two intersecting pits, the mesh is created with 3D elements Solid95, which are well suited for modelling curved boundaries and have good tolerance to irregular shapes without much loss of accuracy. Figure 2a presents the mesh refinement in the region around the pits with the inset showing the whole model meshes. The boundary conditions are specified as follows: as marked in the inset of Figure 2a, the end surface A1 is fully fixed, and a randomly-picked node on the surface A3 is fixed along the Y and Z directions to ensure that no other movement or misalignment occurs. Since the SCF result will not be affected by the magnitude of the load, in all of our analyses, along the X direction a uniformly distributed area load of 1 MPa is applied on the end surface A2 (opposite to A1), and thus the SCF (which is equal to the local maximum stress) can be directly read from the stress contour plot. A linear elastic material model of AA7075-T6 (*σ*_0.2_ = 461.9 MPa) is used with the elastic modulus of 71.7 GPa and the Poisson’s ratio of 0.33. The C-SCF induced by Pit 1 and Pit 2 is expressed with *K*_tcom_, and the SCF induced by the single pit (Pit 1) is denoted as *K*_tsin_. For the convenience of comparison, the normalized SCF is defined as the ratio of the C-SCF to that of the single pit, and is expressed with *K*_tnor_ as the following,

*K*_tnor_ = *K*_tcom_/*K*_tsin_ (2)

To achieve good accuracy of the FE (finite element) solutions, a mesh convergence study is carried out before all of the stress analyses. Concerning the P_de_-P_id_ case with *λ* = 2 and *θ* = 90°, the C-SCF is almost convergent for the model with 180,000 elements, as shown in Figure 2b. Thus in the following studies, all the models are meshed with about 200,000 elements to both ensure the accuracy and avoid extra computational efforts.

## 3. Results and Discussion

### 3.1. The Influence of the Relative Position Parameters

#### 3.1.1. Two Intersecting Pits

**Figure 3:**
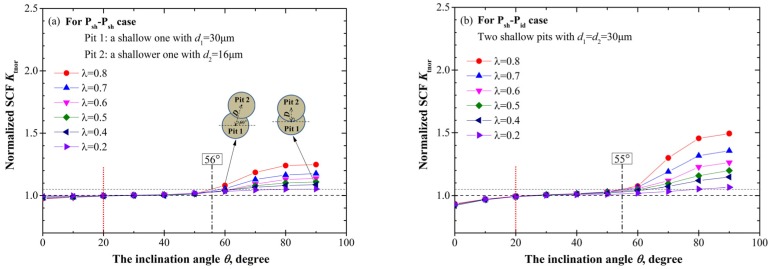

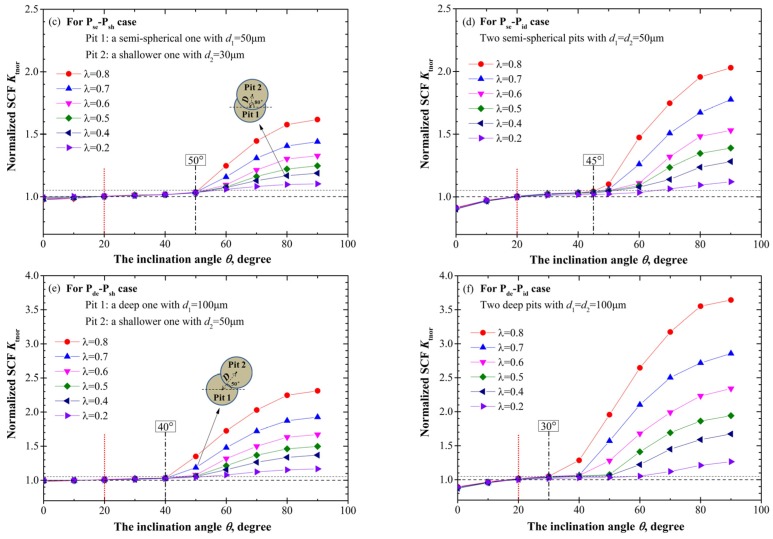
The variation of *K*_tnor_ with the relative position parameters *λ* and *θ* for two intersecting pits. (**a**) P_sh_-P_sh_ case; (**b**) P_sh_-P_id_ case; (**c**) P_se_-P_sh_ case; (**d**) P_se_-P_id_ case; (**e**) P_de_-P_sh_ case; and (**f**) P_de_-P_id_ case.

Figure 3 shows the variation of *K*_tnor_ with the relative position parameters *θ* and *λ* for six intersecting cases. Apparently, for all the cases *K*_tnor_ increases with the increase of the inclination angle *θ* as well as the dimensionless spacing parameter *λ*, and always, *K*_tnor_ reaches its maximum at *θ* = 90° for any *λ* discussed. Here, we define the inclination angle corresponding to the maximum *K*_tnor_ as the critical angle and denote it with *θ*_cri_. Thus, for two intersecting pits the critical angle *θ*_cri_ is always 90°, which is a symmetric location for the two pits with respect to the loading direction.

**Figure 4:**
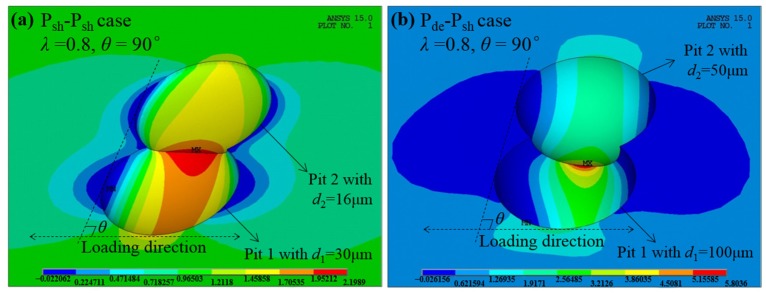
The comparison of stress contours with different pit depths. (**a**) *d*_1_ = 30 μm, *d*_2_ = 16 μm; and (**b**) *d*_1_ = 100 μm, *d*_2_ = 50 μm. The units of stress in this and the subsequent figures are MPa.

By comparing the shallow cases (Figure 3a,b) to the hemi-spherical (Figure 3c,d) and deep cases (Figure 3e,f), it can be found that *K*_tnor_ increases with the increase of the pit depth. For the given spatial parameters *λ* = 0.8 and *θ* = 90°, *K*_tnor_ is about 1.25 for the shallow P_sh_-P_sh_ case (*K*_tcom_ = 2.2), while for the deep P_de_-P_sh_ case *K*_tnor_ increases to about 2.31 (*K*_tcom_ = 5.8). The corresponding stress contours are given and compared in Figure 4, reflecting the higher stress concentration and the greater stress gradient for the deeper pits.

**Figure 5:**
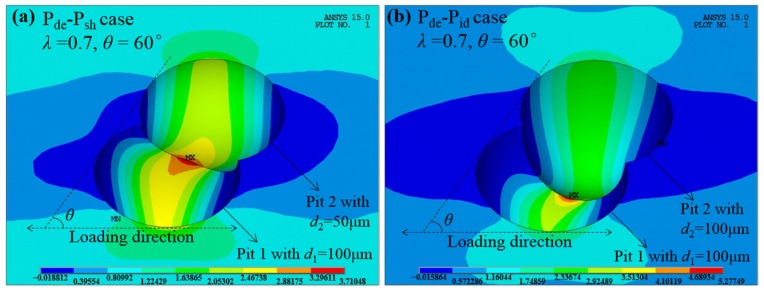
The comparison of stress contours with different depths of Pit 2. (**a**) *d*_1_ = 100 μm, *d*_2_ = 50 μm; and (**b**) *d*_1_ = 100 μm, *d*_2_ = 100 μm.

Furthermore, when comparing Figure 3a,c,e to Figure 3b,d,f respectively, it is clear that interacting effects are more pronounced for the two identical pits than those for the counterparts with a shallower pit, which is further graphically illustrated in Figure 5 with the stress contour of the P_de_-P_sh_ case (*d*_1_ = 100 μm, *d*_2_ = 50 μm) compared to that of the P_de_-P_id_ case (*d*_1_ = 100 μm, *d*_2_ = 100 μm) when *λ* = 0.7 and *θ* = 60°. This feature implies that a neighboring pit whose size is roughly identical to the single pit can exert more influence on the stress field than a shallower one.

Another interesting phenomenon shown in Figure 3 is that *θ* = 20° (marked with the dot lines) looks like a turning point for interacting effects. When *θ* > 20°, *K*_tnor_ is greater than 1, i.e., *K*_tcom_ > *K*_tsin_, which reflects the amplification effect on the stress concentration. When *θ* < 20°, *K*_tnor_ is less than 1, i.e., *K*_tcom_ < *K*_tsin_, which indicates the relaxation effect on the stress concentration. Comparing Figure 3a,c,e to Figure 3b,d,f, the relaxation effect for the shallower cases is insignificant due to the lower influence induced by the shallower neighboring pit, thus *K*_tnor_ approximately remains 1 when *θ* < 20°.

**Figure 6:**
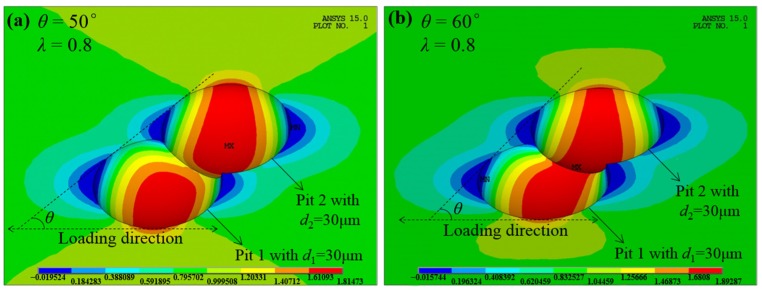
The comparison of stress contours with different inclination angle *θ* for the P_sh_-P_id_ case (*d*_1_ = *d*_2_ = 30 μm) when *λ* = 0.8 and *θ*_th_ = 55°. (**a**) *θ* < *θ*_th_; and (**b**) *θ* > *θ*_th_.

At the initial increase of *θ* from 20°, owing to such a slight growth of *K*_tnor_, we assume that the amplification effect actually begins to work when the C-SCF increases by 5% over that of the single pit, and define the inclination angle corresponding to *K*_tnor_ = 1.05 as the threshold angle *θ*_th_, as marked and labeled in Figure 3 for the limiting case of *λ* = 0.8. The comparison of stress contours with different *θ* is shown in Figure 6 for the P_sh_-P_id_ case (*θ*_th_ = 55°) when *λ* = 0.8. We can see that when *θ* < *θ*_th_ (50° in Figure 6a), the maximum C-SCF is located on the wall of Pit 2, and the stress distribution is almost the same with that of a single pit [4]. Yet when *θ* > *θ*_th_ (60° in Figure 6b), the maximum C-SCF will shift to the common separation wall between the two pits, which means that the neighboring pit begins to exert influence on the combined stress field.

#### 3.1.2. Two Adjacent Pits

**Figure 7:**
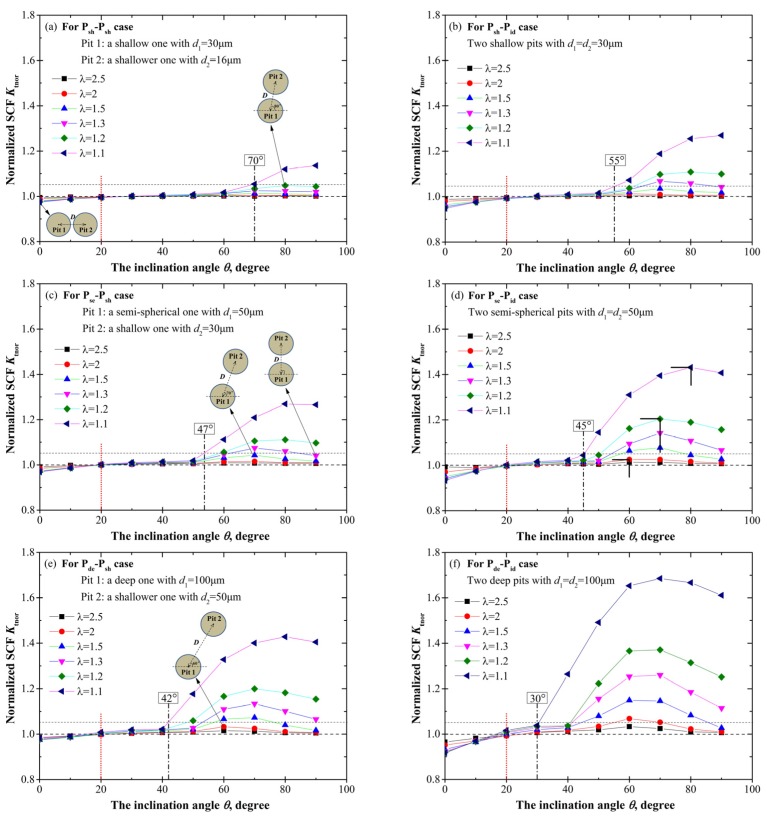
The variation of *K*_tnor_ with the relative position parameters *λ* and *θ* for two adjacent pits. (**a**) P_sh_-P_sh_ case; (**b**) P_sh_-P_id_ case; (**c**) P_se_-P_sh_ case; (**d**) P_se_-P_id_ case; (**e**) P_de_-P_sh_ case; and (**f**) P_de_-P_id_ case.

**Figure 8:**
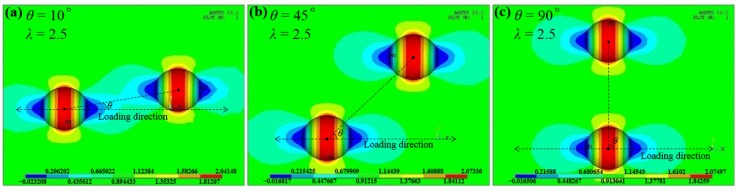
The comparison of stress contours with different *θ* for the P_se_-P_id_ case (*d*_1_ = *d*_2_ = 50 μm) when *λ* = 2.5, showing little interacting effect between these two pits. (**a**) For *θ* = 10°; (**b**) for *θ* = 45°; and (**c**) for *θ* = 90°.

Contrary to intersecting cases, for adjacent pits the normalized SCF *K*_tnor_ decreases with the increase of the dimensionless spacing parameter *λ*, as shown in Figure 7. Moreover, when *λ* is greater than 2, *K*_tnor_ approximately remains 1 irrespective of any variation of *θ*, which is exemplified in Figure 8 with the stress contours at three different *θ* (10°, 45°, 90°) for the P_se_-P_id_ case when *λ* = 2.5.

As shown in Figure 7 and Figure 8, when *λ* > 2, a neighboring pit will have little contribution to the stress concentration resulting from a single pit, which is in good accordance with the similar conclusions proposed for both pits [11,19] and cracks [23,24].

In broad terms, the variation tendency for *K*_tnor_ with the pit depth is similar to that for intersecting cases, but with a less pronounced increase for adjacent pits which can be attributed to the larger spacing between the two pits. In addition, the turning point *θ* = 20° and the threshold value *θ*_th_ exist for adjacent pits as well, and the decreasing trend of *θ*_th_ with the increase of the pit depth is nearly the same with that for intersecting pits. It should be noted that at the limiting case *λ* = 1.1, the *θ*_th_ for two identical shallow, hemi-spherical, and deep pits is 55°, 45°, and 30°, respectively, which correspondingly equals the counterpart for intersecting cases.

**Figure 9:**
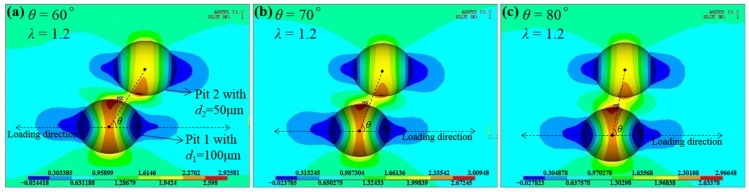
The comparison of stress contours with different *θ* for the P_de_-P_sh_ case (*d*_1_ = 100 μm, *d*_2_ = 50 μm) when *λ* = 1.2, showing the slight asymmetric characteristic that results in the maximum C-SCF (combined stress concentration factor). (**a**) For *θ* = 60°; (**b**) for *θ* = 70°; and (**c**) for *θ* = 80°.

Unlike the monotonous relationship of *K*_tnor_ with *θ* for intersecting cases, for adjacent pits the maximum *K*_tnor_ does not always occur at the symmetric location which may be intuitively expected. Actually, only shallow cases (Figure 7a,b) with *λ* = 1.1 act like intersecting cases, i.e., *K*_tnor_ reaches its maximum at *θ*_cri_ = 90°. For all the other cases shown in Figure 7, *K*_tnor_ reaches its maximum at a slightly asymmetric location, such as *θ*_cri_ = 80° and *θ*_cri_ = 70°. Figure 9 presents the stress contours at *θ*_cri_ = 70° compared to those at *θ* = 60° and *θ* = 80° for the P_de_-P_sh_ case when *λ* = 1.2. It can be found that the maximum stress is located on the wall of Pit 1 near the common separation region, and the C-SCF reaches its maximum at *θ*_cri_ = 70°. It is interesting that the slight asymmetry will result in the maximum C-SCF, which has also been reported for cracks [25] and holes [26], i.e., the interaction effect reaches its maximum in the configuration where the symmetry is slightly perturbed instead of in the ideally symmetric arrangement. Yet the dependence of this asymmetry on the pit depth, as well as the spacing between two pits, has so far not been discussed.

As we can see from Figure 7, for a fixed *λ*, *θ*_cri_ slightly decreases with the increase of the pit depth. Taking two identical pits for an example, when *λ* = 1.1, *θ*_cri_ equals to 90°, 80°, and 70° for double shallow, hemi-spherical, and deep pits, respectively, which can be correspondingly observed in Figure 7b,d,e. The more accurate relationship of *θ*_cri_ and the pit depth will be discussed with more cases below.

**Figure 10:**
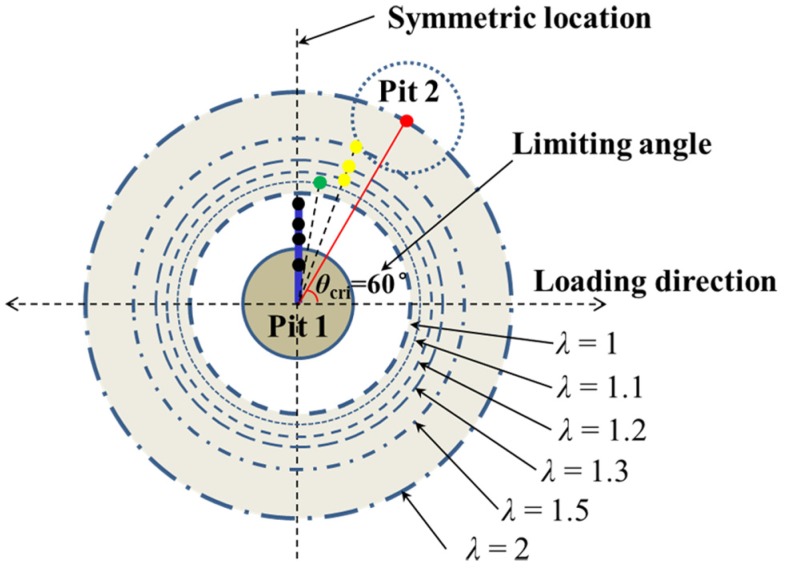
Schematic drawing showing the relationship of *θ*_cri_ with *λ*, reflecting the dependence of the asymmetry feature on the spacing between two pits. For clarity, the locations of Pit 2 are presented with colored dots. The black, green, yellow, and red dots correspond to 90°, 80°, 70° and 60°, respectively.

In addition, with the increasing of *λ* from 1.1 to 2, *θ*_cri_ generally decreases in the range of 60–80°. This tendency is illustrated explicitly with black orthogonal symbols in Figure 7d, that is, *θ*_cri_ = 80° for *λ* = 1.1, *θ*_cri_ = 70° for *λ* = 1.2, 1.3, and 1.5, and *θ*_cri_ = 60° for *λ* = 2. Together with the results for the intersecting pits (i.e., *θ*_cri_ = 90° for 0 < *λ* < 1), the variation tendency of *θ*_cri_ with *λ* can be schematically illustrated in Figure 10. The colored dot is used to point out the location of Pit 2. We can see that with the increase of the spacing between the two pits, the C-SCF will reach its maximum at *θ* deviating more from the symmetric location, and notably, the limiting angle will be *θ*_cri_ = 60° since there is almost no interacting effects between the two pits when *λ* > 2.

#### 3.1.3. The Critical Corrosion Region

**Figure 11:**
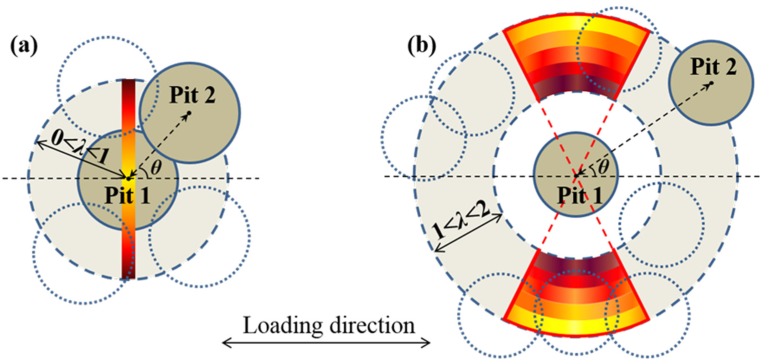
Diagram showing the critical corrosion region for a neighboring pit. The dashed circle represents the possible location for Pit 2, and the colored area corresponds to the critical corrosion region, with the darker color illustrating the higher C-SCF. (**a**) For intersecting pits; and (**b**) for adjacent pits. Notably, for a fixed *λ* in Figure 11b, the C-SCF slightly varies with the inclination angle *θ*, which is schematically illustrated with the color gradients in each stained band.

According to the severity of the C-SCF, we can summarize the influence of the relative position parameters and determine the critical corrosion region for Pit 2, which denotes the area with high susceptibility to fatigue damage due to the amplified C-SCF. Figure 11 gives the schematic illustration displaying the critical corrosion regions for both the intersecting and adjacent cases, where the dashed circle represents the possible location for Pit 2. With regard to the intersecting pits (0 < *λ* < 1), the C-SCF reaches its maximum at *θ*_cri_ = 90°, thus the colored band in Figure 11a corresponds to the critical corrosion region for Pit 2. While for adjacent cases (1 < *λ* ≤ 2), the maximum C-SCF occurs in the range of 60° ≤ *θ* ≤ 90° due to the asymmetric characteristic of *θ*_cri_, thus the colored fan-shaped area in Figure 11b gives the critical corrosion region for Pit 2. It is worthwhile to mention that for the colored area, generally, the darker the stained band is, the higher the C-SCF is.

**Figure 12:**
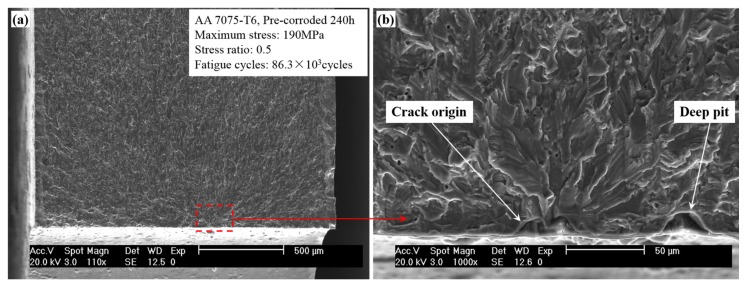
Typical SEM fractography with the fatigue crack initiating from two intersecting pits rather than the deeper pit. (**a**) Overall view of the fracture surface; and (**b**) local view of the crack origin.

The obtained results for *K*_tnor_’s variation with the relative position parameters may provide a mechanics-based explanation for the candidate locations of fatigue crack initiation. Medina et al. [27] observed in the experiments that maximum pit depth showed very low correlation with the location of fatigue fracture. They ascribed this phenomenon to the relaxation of the stress concentration caused by neighboring pits. With reference to our results, we believe that besides the reason mentioned in [27], the amplification effect on the stress concentration is another possible cause for this phenomenon. Figure 12 shows a typical fracture observed through scanning electron microscope (SEM) in our previous work [17]. The fatigue crack initiates from two intersecting pits with *θ* = 90° rather than the nearby deep pit, which can be attributed to the amplification effect on the combined stress concentration.

Furthermore, studies [22,28,29,30] have frequently mentioned that fatigue cracks originate from shallow pits rather than deep ones, and so far there is still no sound explanation for this phenomenon. According to our results, this observation may be attributed to the presence of an adjacent pit located in the critical region, especially when *θ* is in the range of 60°–70°, where the pit may remain invisible during conventional fracture analysis. A neighboring pit like that will induce such an increase in the C-SCF that even a shallow pit, that seems like a single one via SEM observation, can nucleate a fatigue crack and eventually lead to structural failure. This feature also implies that the conventional 2D SEM analysis is not enough to characterize the corrosion fatigue fracture due to interacting effects caused by neighboring pits, and 3D fracture analysis instead should be performed for better understanding of failure mechanisms.

### 3.2. The Influence of Pit Depth

As discussed before, the normalized SCF *K*_tnor_ is not only associated with the relative position parameters *λ* and *θ*, but also with the pit depth *d*. Based on the analysis in Section 3.1, the general characteristics of *K*_tnor_’s variation with the pit depth have been initially observed by focusing on three settings, i.e., shallow, sphere, and deep pits. To further clarify the influence of the pit depth on the C-SCF, we append more FEA calculations with other pit depths regarding two identical neighboring pits. In addition, a dimensionless depth parameter *R*_d_ = *d*/*r*, also called the aspect ratio, is used in the following analysis for the purpose of generality.

#### 3.2.1. The Relationship between *θ*_th_ and Pit Depth

As mentioned in Section 3.1.2, the threshold angle *θ*_th_ generally shows a decreasing trend with the increase of the pit depth, that is, *θ*_th_ is 55°, 45°, and 30° for double shallow (*d* = 30 μm), hemi-spherical (*d* = 50 μm), and deep pits (*d* = 100 μm), respectively. To further confirm this tendency, we perform FEA calculations at other depths with the limiting case of *λ* = 1.1, and determine the threshold angle *θ*_th_ according to the definition in Section 3.1.1. The calculation results are listed in Table 2 with the newly obtained *θ*_th_ shown in bold. It is noteworthy that the *θ*_th_ approaches 90° with the decrease of the pit depth, which suggests that the effect of neighboring pits on the stress concentration is nearly negligible for very shallow pits.

**Figure 13:**
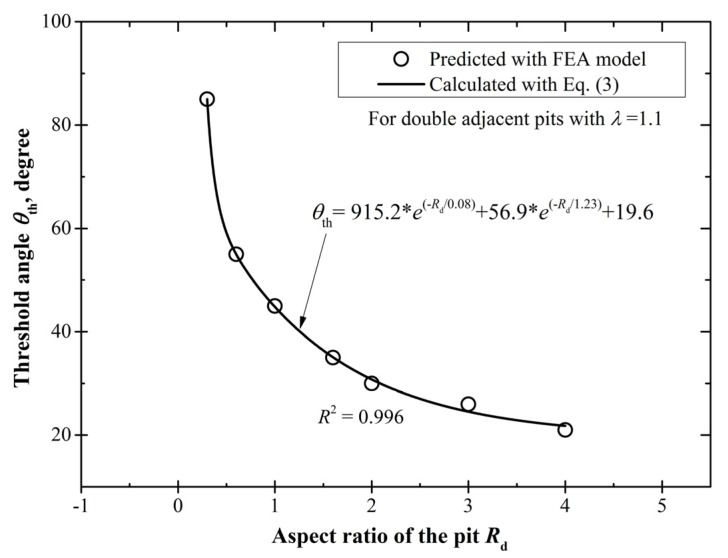
The relationship of the threshold angle *θ*_th_ with the aspect ratio *R*_d_, reflecting the dependence of the affected spatial scope on the pit depth.

Figure 13 shows the decreasing tendency of *θ*_th_ with the increase of *R*_d_, and their relationship can be expressed with an exponential decay function as,

(3) $$\theta_{\mathrm{th}}=915.2\times e^{\left(-\;\frac{R_{d}}{0.08}\right)}\;+\;56.9\times e^{\left(-\frac{R_{d}}{1.23}\right)}\;+\;19.6,0<R_{d}\leq 4$$

Thus, when pit dimensions are obtained, the threshold angle *θ*_th_ can be conservatively estimated with Equation (3) since it is proposed regarding the limiting case of *λ* = 1.1.

#### 3.2.2. The Relationship between *K*_tnor_ and Pit Depth

To better comprehend the dependence of *K*_tnor_ on the pit depth, supplementary calculations are performed for deeper pits with *R*_d_ = 1.6, 3, and 4 at the most disadvantaged configurations (i.e., the high stress concentration) of relative position parameters. Based on the results obtained in Section 3.1.1, for intersecting pits the most disadvantaged configuration is *θ* = 90° with *λ* = 0.8. All results of *K*_tnor_ at diverse pit depths are listed in Table 3 with the newly obtained results shown in bold.

For adjacent cases, the most disadvantaged spacing parameter is clearly *λ* = 1.1. Yet *θ*_cri_ varies with the pit depth, as initially discussed in Section 3.1.2. Thus, we add more FEA calculations for deeper pits in order to find not only the dependence of *θ*_cri_ on the pit depth, but also the variation of *K*_tnor_ with the pit depth. Table 4 lists the newly obtained results (in bold) as well as the results already obtained for *d* = 30 μm, 50 μm, and 100 μm. It can be found that for deeper pits, the maximum *K*_tnor_ always occurs at *θ*_cri_ = 70°. Since there is a slight difference of *K*_tnor_ between *θ*_cri_ = 90° and *θ* = 70° for the P_sh_-P_id_ case (1.27 vs. 1.19), as well as that between *θ*_cri_ = 80° and *θ* = 70° for the P_se_-P_id_ case (1.43 vs. 1.39), we choose the case of *λ* = 1.1 with *θ* = 70° as the most disadvantageous configuration for two adjacent pits.

**Figure 14:**
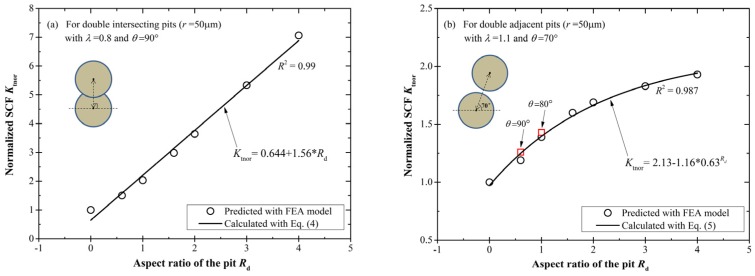
The relationship of the normalized SCF *K*_tnor_ with the aspect ratio *R*_d_ at the most disadvantageous configuration (i.e., the high stress concentration) of the relative position parameters. (**a**) For intersecting pits; and (**b**) for adjacent pits.

Figure 14 shows the relationship of the normalized SCF *K*_tnor_ vs. the aspect ratio *R*_d_ at the most disadvantageous configuration of the relative position parameters. For the intersecting case with *λ* = 0.8 and *θ* = 90° (Figure 14a), *K*_tnor_ presents good linearity with *R*_d_ and the linear growth can be given as,

(4) $$K_{\mathrm{tnor}}\;=\;0.644+1.56\times R_{d},0<R_{d}\leq 4$$

This linear relationship reveals the strong dependence of *K*_tnor_ on the pit depth, and also implies that for intersecting cases, the pit depth is a highly sensitive parameter to which much attention should be paid. However, for the adjacent case with *λ* = 1.1 and *θ* = 70° (Figure 14b), *K*_tnor_ increases exponentially with *R*_d_, and can be written as,

(5) $$K_{\mathrm{tnor}}\;=\;2.13-1.16\times 0.63^{R_{d}},0<R_{d}\leq 4$$

A declining tendency of the amplification effect can be observed with the increase of the pit depth, which suggests the weakened influence of the pit depth on the C-SCF for deeper adjacent pits.

In view of the above discussions, once we acquire the dimensions related to the pits, we can determine whether the interacting effects work on the stress field induced by the neighboring pits. Meanwhile, via the empirical formulas of Equations (4) or (5), we can conservatively estimate the combined SCF because they are proposed regarding to the most disadvantageous configuration of the relative position parameters. This convenient method to estimate the C-SCF is important for the structural safety assessment as well as for the structural design.

## 4. Application

### 4.1. Estimation Procedure for the C-SCF

**Figure 15:**
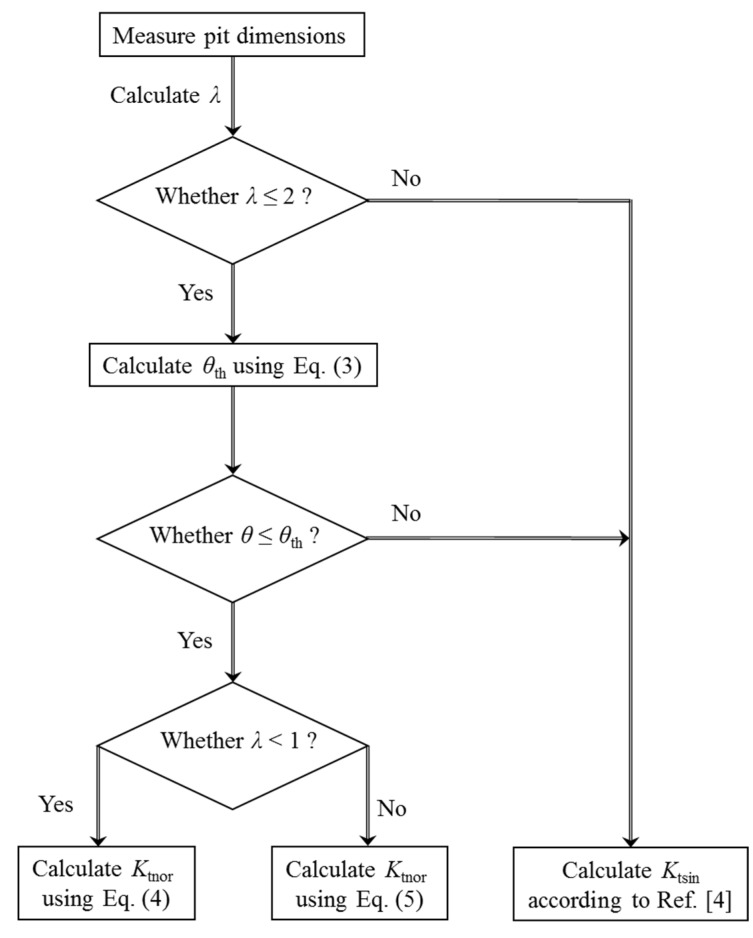
Flow chart for estimating the normalized SCF.

Figure 15 presents the flow chart to conservatively estimate the normalized SCF resulting from two neighboring pits. Firstly, the corrosion morphology is characterized to the obtain pit dimensions. Secondly, the dimensionless spacing parameter *λ* is calculated. If *λ* > 2, there is no significant interacting effect between these two pits, and it is recommended to estimate the SCF according to our previous work [4]. If *λ* ≤ 2, *θ*_th_ should be calculated using Equation (3). The third step is to examine whether the inclination angle *θ* of the two pits is greater than *θ*_th_. Based on the discussion in Section 3.1.1, no interacting effect exists for *θ* < *θ*_th_, and the SCF can be estimated according to [4]. If *θ* > *θ*_th_, the spacing parameter *λ* should be further considered. If 0 < *λ* < 1, we calculate *K*_tnor_ by using the proposed formula of Equation (4), and otherwise, we predict *K*_tnor_ by using the proposed formula of Equation (5). Finally, the C-SCF *K*_tcom_ can be obtained through multiplying *K*_tnor_ by *K*_tsin_, and the latter can be calculated using the method in [4].

### 4.2. Application Example

Full immersion corrosion tests are performed on an aluminum alloy 7075-T6 specimen in 3.5 wt % NaCl aqueous solution for 120 h. After pre-corrosion, the specimens are carefully rinsed in running water for 10 min and then dried.

**Figure 16:**
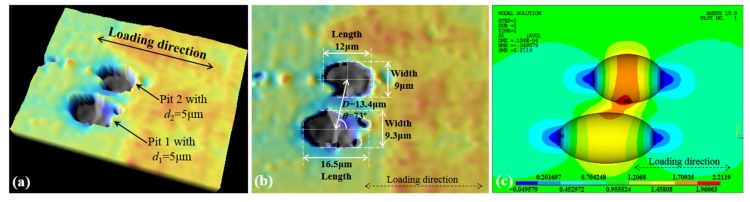
An example for corrosion characterization and the FEA (finite element analysis) result of the C-SCF. (**a**) 3D morphology; (**b**) 2D morphology with pit dimensions; and (**c**) the FEA result for the C-SCF.

With a white light confocal microscope, two neighboring pits are characterized, and the obtained 3D and 2D morphology, as well as their pit dimensions, are shown in Figure 16a,b, respectively. In accordance with the measured dimensions, the FEA model is developed and the C-SCF is calculated. The stress contour is shown in Figure 16c, and the FEA calculation result for *K*_tcom_ is about 2.21.

To conservatively estimate the C-SCF, the elliptical pit is idealized as the one with a circular surface shape, i.e., *r* = 4.5 μm, since the latter has a slightly higher stress concentration effect than the former [4]. According to the measured dimensions, the dimensionless spacing parameter is *λ* = 13.4/9 ≈ 1.5, and is evidently less than 2. Then, we calculate the critical angle *θ*_th_ for these two pits by using Equation (3), and the result is *θ*_th_ = 43° with the aspect ratio *R*_d_ = 5/4.5 ≈ 1.11. As shown in Figure 16b, the measured inclination angle *θ* is 73°, and is apparently larger than *θ*_th_. Therefore, the interacting effects will work on the stress field induced by Pit 1 and Pit 2, and the normalized SCF *K*_tnor_ can be estimated with Equation (5) proposed for two adjacent pits. By substituting *R*_d_ into the empirical formula, the achieved *K*_tnor_ is about 1.43.

With reference to our previous work [4], the SCF induced by a single pit (*K*_tsin_) is about 2.12, thus the C-SCF can be predicted as the following,

*K*_tcom_ = *K*_tnor_ × *K*_tsin_ = 1.43 × 2.12 ≈ 3.03. (6)

Compared to the FEA result (~2.21), the estimation error factor is about 1.37, which is mainly attributed to the conservative *K*_tnor_ calculated according to the most disadvantageous configuration of the relative position parameters. Additionally, the pit idealization, assuming the elliptical pit as the circular one, also introduces errors into the calculation of *K*_tsin_ and results in a larger value than the actual one (2.12 vs. 1.83 for the elliptical pit). The error factor of 1.37 may seem over-conservative. However, for circumstances where in-situ observations of pit dimensions are impossible or with worse accuracy, a relatively conservative estimation is usually needed and feasible for safety assessments. Therefore, the estimation method proposed here is still highly recommended given that a safety margin is always required to ensure the reliability of fatigue-related analysis and design.

## 5. Conclusions

By comprehensively considering the pit depth and the relative position parameters of two neighboring pits, their interacting effects on the stress concentration are analyzed and discussed. The noteworthy findings are summarized as follows,

(1) For two intersecting pits, the combined SCF *K*_tcom_ increases with the increase of the relative position parameters *θ* and *λ*, and *K*_tcom_ always reaches its maximum at the symmetric location (*θ* = 90°) in the range of 0 < *λ* <1.
(2) For two adjacent pits, the combined SCF *K*_tcom_ decreases with the increase of *λ*, and when *λ* > 2, the interacting effects have little contribution to the combined SCF, thus *K*_tcom_ ≈ *K*_tsin_. The maximum C-SCF occurs at the asymmetric location of 60° ≤ *θ*_cri_ ≤ 90°. Meanwhile, *θ*_cri_ decreases with the increase of *λ* from 1 to 2, and *θ*_cri_ = 60° can be regarded as the limiting angle of this asymmetric feature.
(3) The threshold angle *θ*_th_ exponentially decreases with the aspect ratio *R*_d_ (Equation (3)), which implies that the interacting effects induced by two neighboring pits exert influence in a larger scope for the deeper pits.
(4) The normalized SCF generally increases with the pit depth, and follows a linear and an exponential relationship with *R*_d_ for two intersecting and adjacent pits, respectively. For the most disadvantageous configurations (i.e., the high stress concentration) of the relative position parameters, the empirical formulas (Equations (4) and (5)) are proposed to conservatively estimate the combined SCF and are validated via a practical example with the estimation error factor of 1.37.

In future work, pit eccentricity should be taken into account to further elucidate the interacting effect between two neighboring pits. In addition, a more complete study concerning a random arrangement of multi-pits will also be an interesting investigation.

## Figures and Tables

**Table 1:** The definitions and settings for the configurations of Pit 1 and Pit 2.

Definition		Pit 2 (μm)	
		Shallower	Identical
**Pit 1 (μm)**	Shallow	P_sh_-P_sh_ (*d*_1_ = 30, *d*_2_ = 16)	P_sh_-P_id_ (*d*_1_ = 30, *d*_2_ = 30)
	Hemi-spherical	P_se_-P_sh_ (*d*_1_ = 50, *d*_2_ = 30)	P_se_-P_id_ (*d*_1_ = 50, *d*_2_ = 50)
	Deep	P_de_-P_sh_ (*d*_1_ = 100, *d*_2_ = 50)	P_de_-P_id_ (*d*_1_ = 100, *d*_2_ = 100)

**Table 2:** The results of *θ*_th_ for double adjacent pits (*λ* = 1.1) with various pit depths. The newly obtained ones are presented in bold.

*d* (μm)	*R*_d_	*K*_tsin_	*K*_tnor_	*θ*
**15**	**0.3**	**1.53**	1.051	86°
			**1.047**	**85°**
30	0.6	1.76	1.05	55°
50	1	2.06	1.05	45°
**80**	**1.6**	**2.37**	1.057	36°
			**1.033**	**35°**
100	2	2.51	1.05	30°
**150**	**3**	**2.72**	1.051	27°
			**1.048**	**26°**
**200**	**4**	**2.82**	1.052	22°
			**1.046**	**21°**

**Table 3:** The results of *K*_tnor_ at diverse pit depths for double intersecting pits with *λ* = 0.8 and *θ* = 90°. The supplementary results are shown in bold.

*d* (μm)	*R*_d_	*K*_tsin_	*K*_tnor_
30	0.6	1.76	1.49
50	1	2.06	2.03
**80**	**1.6**	**2.37**	**2.98**
100	2	2.51	3.64
**150**	**3**	**2.72**	**5.33**
**200**	**4**	**2.82**	**7.062**

**Table 4:** The variation of *K*_tnor_ and *θ*_cri_ with the pit depths for double adjacent pits when *λ* = 1.1. The supplementary results are listed in bold.

*d* (μm)	*R*_d_	*K*_tsin_	*K*_tnor_	*θ*
30	0.6	1.76	1.27	90°
			1.19	70°
50	1	2.06	1.43	80°
			1.39	70°
**80**	**1.6**	**2.37**	1.591	75°
			**1.602**	**70°**
			1.561	65°
100	2	2.51	1.69	70°
**150**	**3**	**2.72**	1.782	72°
			**1.83**	**70°**
			1.786	65°
**200**	**4**	**2.82**	1.89	72°
			**1.934**	**70°**
			1.877	65°
